# Mixed large cell neuroendocrine carcinoma and adenocarcinoma of the gallbladder: a case report and brief review of the literature

**DOI:** 10.1186/s12957-015-0533-6

**Published:** 2015-03-18

**Authors:** Wei Liu, Lei Wang, Xiao-dong He, Cheng Feng, Xiao-yan Chang, Zhao-hui Lu

**Affiliations:** Department of General Surgery, Peking Union Medical College Hospital, Chinese Academy of Medical Sciences and Peking Union Medical College, 1# Shuaifuyuan, Dongcheng District Beijing, 100730 China; Peking University Wu-Jieping Urology Center, Peking University Shougang Hospital, Peking University Health Science Center, 9# Jinyuanzhuang Road, Shijingshan District Beijing, 100144 China; Department of Pathology, Peking Union Medical College Hospital, Chinese Academy of Medical Sciences and Peking Union Medical College, 1# Shuaifuyuan, Dongcheng District Beijing, 100730 China

**Keywords:** Cholecyst, Neuroendocrine tumors, Adenocarcinoma, Neoplasms

## Abstract

Large-cell neuroendocrine carcinoma (LCNEC) of the gallbladder is extremely rare. We present a 63-year-old Chinese female who was admitted with right upper quadrant pain and a quasi-circular tumor measuring 2.0 cm on the body of the gallbladder, as indicated by computed tomography. LCNEC combined with adenocarcinoma was immunohistochemically confirmed after open radical cholecystectomy. Postoperative recovery of this patient was uneventful, and no evidence of recurrence or metastasis was observed after 12 months of follow-up. LCNEC of the gallbladder is thought to be extremely rare and is usually found in combination with other histological carcinoma types, such as adenocarcinoma, as determined histologically. The prognosis is poor overall, but early detection with complete resection may result in a relatively good prognosis.

## Background

Neuroendocrine neoplasms (NENs) account for 1.25% of all malignancies, with the majority (66%) occurring in the gastrointestinal tract, followed by the bronchopulmonary system (31%). Other less frequent locations include the ovaries, testes, pancreas, and hepatobiliary system. Gallbladder NENs are particularly rare, and 278 cases were registered in the Surveillance, Epidemiology and End Results (SEER) from 1973 to 2005, only representing 0.5% of all NENs and approximately 2% of all gallbladder cancers [1,2]. Among all NENs originating in the gallbladder, large-cell neuroendocrine carcinoma (LCNEC) is exceedingly rare, and the first case worldwide was reported in 2000 [3]. To date, although several case reports of gallbladder LCNEC have been published [3-16] (Table 1), its biological behavior, the appropriate treatment modalities, and the overall patient prognosis remain largely unclear. Herein, we report a rare case of gallbladder LCNEC and present a brief literature review to contribute to the increased understanding of the clinical features of this disease.

**Table 1:** **Clinicopathological features of 17 cases of large**-**cell neuroendocrine carcinoma of the gallbladder**

**Number**	**Author** **[ref.]**	**Gender/** **age**	**Presentation**	**Tumor location**	**Tumor size** **(cm)**	**Liver invasion**	**Metastasis**	**Other component**	**Treatments**	**Prognosis**, **follow**-**up** **(month)**
1	Papotti *et al*. [3]	M/50	Unclear	Unclear	<1	-	-	AC	Cho	DFS, 12
2	Papotti *et al*. [3]	M/65	Unclear	Fundus	2.5	-	Liver	-	Cho	Died, 14
3	Jun *et al*. [4]	M/55	AP, jaundice	Unclear	Unclear	Unclear	Lymph node	-	Needle biopsy, Che	Died, 1
4	Jun *et al*. [4]	F/67	AP	Unclear	Huge	+	Lymph node	-	Needle biopsy, Che	Died, 10
5	Noske and Pahl [5]	F/81	AP, jaundice	Neck	5	+	Bone	Adenosquamous	Palliative surgery	Unknown
6	Oshiro *et al*. [6]	F/55	Back pain, fever	Body	4.9	-	-	AC, SCNEC	Radical Cho	DFS, 20
7	Shimono *et al*. [7]	F/64	AP	Unclear	11.5	+	-	-	Che, Rad, extended hepatectomy	DFS, 36
8	Iype *et al*. [8]	M/85	Anorexia, weight loss	Fundus	1.5	-	Unclear	AC	Cho, Che	Died, 21
9	Lin *et al*. [9]	F/65	Cushing’s syndrome	Body	Unclear	-	-	-(ACTH-producing)	Radical Cho, Che	Died, 2
10	Sato *et al*. [10]	F/68	Negative	Fundus	3	+	Lymph node	AC	Cho, extended hepatectomy	DFS, 12
11	Paniz *et al*. [11]	F/48	AP	Fundus	3.5	+	Unclear	AC	Cho, extended hepatectomy	Unknown
12	Al-Brahim and Albannai [12]	M/45	AP, jaundice	Fundus	5.7	+	Unclear	AC	Cho, Che	Unknown
13	Okuyam *et al*. [13]	M/64	Abdominal fullness	Fundus	2.5	+	Lymph node	-	Biopsy, Che	Died, 22
14	Nakagawa *et al*. [14]	M/56	Exophthalmos	Unclear	9	+	Multiple	AC	Che, Rad	Died, 36
15	Meguro *et al*. [15]	F/54	Negative	Unclear	Unclear	-	-	AC	Cho, extrahepatic bile duct resection	DFS, 24
16	Russo *et al*. [16]	M/59	AP	Body	4	+	Lymph node	Mucinous carcinoma	Radical Cho	Unknown
17	Current study, 2014	F/63	AP, fever	Body	2.0	-	-	AC	Radical Cho	DFS, 12

AP, abdominal pain; AC, adenocarcinoma; ACTH, adrenocorticotropic hormone; Che, chemotherapy; Cho, cholecystectomy; DFS, disease-free survival; F, female; M, male; Rad, radiotherapy; SCNEC, small-cell neuroendocrine carcinoma.

## Case presentation

**Figure 1:**
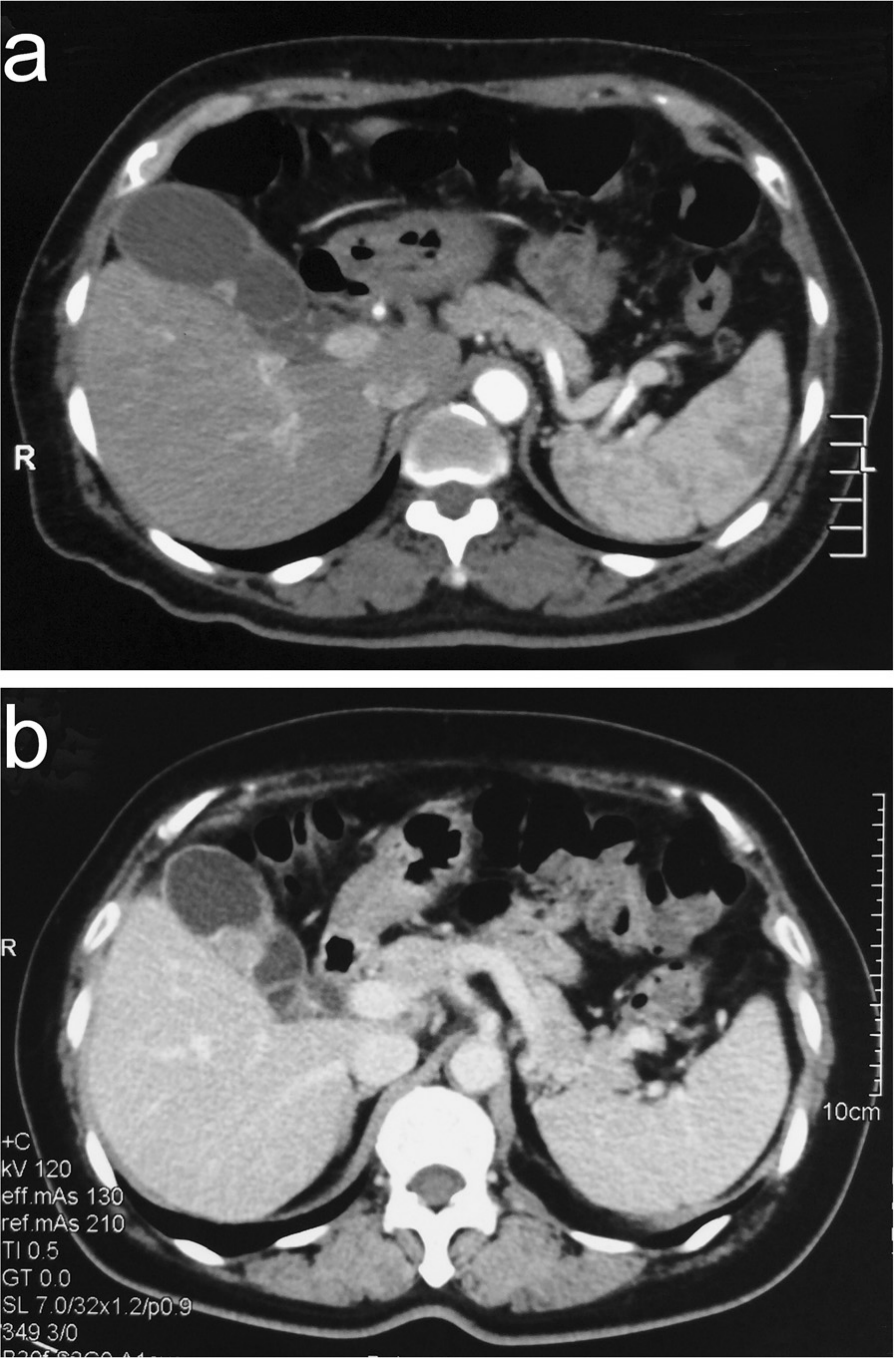
**A fast**-**growing tumor located on the body of the gallbladder. (a)** Computed tomography showed a suspected mass, measuring 0.6 cm, on the gallbladder plica 3.5 months prior. **(b)** At admission 3.5 months later, CT showed a 2.0 × 1.8 cm quasi-circular mass located on the body of gallbladder, with significant enhancement in the portal venous phase.

A 63-year-old woman came to our emergency room on 14 July 2013, with a history of intermittent right upper quadrant pain that began 3 months prior. Ultrasound indicated no other anomalies, except for a focal thickening of the gallbladder wall. Computed tomography (CT) showed a suspected mass measuring 0.6 × 0.3 cm on the gallbladder plica (Figure 1a). Because she had a past medical history of cholecystitis, we could not clearly determine whether the mass was a tumor or only thickened mucosa; thus, watchful follow-up was recommended to the patient. On the 29^*th*^ of October 2013, the patient returned to our clinic, and CT scan revealed a 2.0 × 1.8 cm quasi-circular tumor located on the body of the gallbladder in the same location as the mass detected 3.5 months earlier, with significant enhancement in the portal venous phase (Figure 1b). The results of laboratory tests, including tests for tumor markers and hormonal profiles, were all within normal limits.

**Figure 2:**
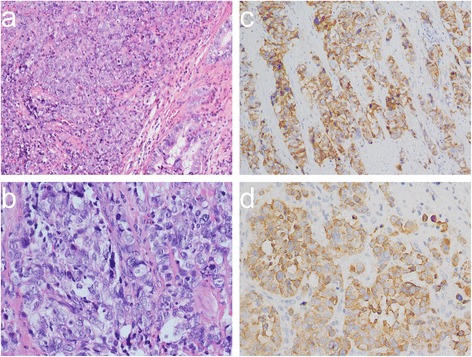
**Pathologically demonstrated mixed large**-**cell neuroendocrine carcinoma and adenocarcinoma of the gallbladder. (a)** A low-power view (H&E, 100×) demonstrating the combination of a majority of poorly differentiated large cell neuroendocrine carcinoma (LCNEC) cells and a minority of moderately differentiated adenocarcinoma cells (right lower quadrant). **(b)** A high-power view (H&E, 400×) demonstrating that the neuroendocrine carcinoma cells were large in size, polygonal, and contained high numbers of mitotic figures. **(c)** Immunohistochemical staining showing that the LCNEC cells were positive for chromogranin A. **(d)** Immunohistochemical staining showing that the LCNEC cells were positive for synaptophysin.

Laparoscopic cholecystectomy was performed, and an intraoperative frozen pathological section indicated that the lesion was malignant. Immediately thereafter, open radical cholecystectomy with resection of a wedge of the liver and the hepatoduodenal lymph nodes was performed. The gross specimen showed a cauliflower-shaped mass, and microscopically, the tumor consisted of the following two components: moderately differentiated adenocarcinoma and poorly differentiated large-cell neuroendocrine carcinoma (Figure 2a,b). Immunohistochemically, the neuroendocrine cells exhibited the strong expression of the neuroendocrine markers chromogranin A (Figure 2c) and synaptophysin (Figure 2d). In addition, these neuroendocrine cells showed a Ki67 index of over 80%. There was no evidence of serous or liver invasion or lymph node or distant metastasis. Thus, this lesion was assigned a final classification of pT2N0M0 stage II, according to the Union Internationale Contre le Cancer guidelines. The postoperative course of this patient was uneventful, and the carcinoma did not recur during a 12-month follow-up period.

The present case report is in compliance with the Helsinki Declaration and has been approved by ethics committee of Peking Union Medical College Hospital.

## Discussion

According to the latest World Health Organization (WHO) classification published in 2010 [17], NENs are classified into the following four general categories that are mainly based on mitotic count and the Ki67 proliferation index: (1) well differentiated neuroendocrine tumor (NET) or grade 1 tumor, with a mitotic count of <2/10 per high-power fields (HPF) and a Ki67 of ≤2%, such as a typical carcinoids; (2) intermediate differentiated NET or grade 2 tumor, with a mitotic count of between 2 and 20/10 HPF and a Ki67 of 3% to 20%, such as an atypical carcinoids; (3) poorly differentiated neuroendocrine carcinoma (NEC) or grade 3 tumor, with a mitotic count of >20/10 HPF and a Ki67 of >20%, which includes small-cell and large-cell NECs; and (4) mixed adenoneuroendocrine carcinoma (MANEC), histologically exhibiting concomitant adenocarcinoma (or other components) and NEC concomitantly. Primary gallbladder small-cell NEC (GB-SCNEC) is particularly rare, with only 74 cases described until 2011 [18]. Large-cell neuroendocrine carcinoma of the gallbladder (GB-LCNEC) is exceedingly rare and was first reported by Papotti in 2000 [3]. The histological features of LCNEC are as follows: (1) positivity for neuroendocrine markers, among which chromogranin A and synaptophysin are the most commonly identified; (2) a mitotic count exceeding 20/10 HPFs or a Ki67 index of over 20%; and (3) a specific NET pattern of an organoid structure, rosette formation, palisading, and trabecular arrangement, as well as prominent nuclei that are over three times the diameter of a lymphocyte. Although more than ten cases of GB-LCNEC have been reported in the English literature to date (Table 1), there is a paucity of data on this tumor type.

We reviewed a series of 17 GB-LCNECs, including 16 previously reported cases and our present case. This series of GB-LCNECs included reports of 6 (35%) pure LCNECs and 11 (65%) LCNECs combined with other histological components, including 9 concomitant with adeno-, one with adenosquamous-, and one with mucinous carcinoma. Patients with mixed histological components were classified as having MANEC according to the WHO 2010 classification [17]. Only one tumor was found to be a functional ACTH-producing tumor in this series.

Enterochromaffin cells, the precursor cells of NENs, are distributed throughout the gastrointestinal tract, bronchus, endocrine glands, and skin. These are also common sites of NEN. The gallbladder mucosa in the fundus and body is devoid of neuroendocrine cells, which may appear after intestinal metaplasia due to chronic inflammation. This fact explains why NENs rarely occur in the gallbladder. Virtually all published reports on gallbladder NENs describe coexisting gallstones and chronic cholecystitis [19]. Our patient presented non-specific vague abdominal pain in the right upper quadrant, which is a typical symptom of cholelithasis and should be considered a pathogenic basis of NEC.

Similar to most previously reported cases of NEC, the clinical symptoms and radiological findings of our patient were nonspecific. According to the descriptions of the series of 17 GB-LCNECs, abdominal pain (8/17, 47%) was the most common symptom. Other symptoms, including fever, jaundice, and weight loss, were also nonspecific. SCNEC and LCNEC were confirmed to be genetically similar and distinct from well-differentiated NETs [20]. LCNEC was thought to exhibit similar aggressive behavior and early metastasis compared with SCNEC [4]. Among the 17 GB-LCNECs described, nine (53%) were diagnosed with direct hepatic invasion, and eight (47%) were identified with metastasis in the lymph nodes, liver, or bone. Fortunately, our case showed no signs of liver invasion or distant metastasis, which may have been attributed to early detection.

Surgical resection, which has been determined to improve the prognosis of patients with NEC [21], is considered to be the main treatment for gallbladder NECs. The prognosis is very poor for patients with unresectable masses [22], although multimodal treatments, including chemotherapy and radiation therapy, have achieved good responses in some reports [7]. Follow-up results were reported for 13 of the 17. Among them, seven (54%) died of the disease in a median time of 14 months, while the other six patients exhibited disease-free survival (DFS) after 12 to 36 months of follow-up. Four surviving patients had some similar characteristics, such as small tumor size and no liver invasion or metastasis, and they had undergone radical surgery. The other two successful treatments for patients with liver invasion or lymph node metastasis mainly rely on extended surgery with hepatectomy and lymph node dissection. The presence of the adenocarcinoma phenotype is thought to indicate poor prognosis [2]. However, we could not make a similar conclusion according to this series of the 17 GB-LCNECs.

## Conclusions

LCNEC of the gall bladder is extremely rare and is usually present along with other carcinoma types, such as adenocarcinoma, as determined histologically. The patient prognosis is poor overall, but early detection with complete resection may result in a relatively good prognosis.

## Consent

Written informed consent was obtained from the patient for publication of this case report and any accompanying images. A copy of the written consent is available for review by the editor of this journal.

## Abbreviations

CT: computed tomography.
GB-LCNEC: large-cell neuroendocrine carcinoma of the gallbladder
GB-SCNEC: small-cell neuroendocrine carcinoma of the gallbladder
LCNEC: large-cell neuroendocrine carcinoma
MANEC: mixed adenoneuroendocrine carcinoma
NEC: neuroendocrine carcinoma
NEN: neuroendocrine neoplasm
NET: neuroendocrine tumor
SCNEC: small-cell neuroendocrine carcinomas
